# Transcriptional regulator PrqR plays a negative role in glucose metabolism and oxidative stress acclimation in *Synechocystis* sp. PCC 6803

**DOI:** 10.1038/srep32507

**Published:** 2016-09-01

**Authors:** Rezaul Islam Khan, Yushu Wang, Shajia Afrin, Bing Wang, Yumin Liu, Xiaoqing Zhang, Lei Chen, Weiwen Zhang, Lin He, Gang Ma

**Affiliations:** 1 Bio-X Institutes, Key Laboratory for the Genetics of Developmental and Neuropsychiatric Disorders (Ministry of Education), Shanghai Jiao Tong University, Shanghai 200240, P.R. China; 2 School of Electronics and Information Engineering, Tongji University, Shanghai 201804, China; 3 Instrumental Analysis Center of Shanghai Jiao Tong University, Shanghai 200240, P.R. China; 4 Laboratory of Synthetic Microbiology, School of Chemical Engineering & Technology, Tianjin University, Tianjin 300072, P.R. China; 5 Collaborative Innovation Center of Chemical Science and Engineering, Tianjin, P.R. China

## Abstract

Plant and cyanobacteria can perceive signals from soluble sugar and reactive oxygen species (ROS) and then coordinate gene expression under stress acclimation, but the underlying mechanism remains unclear. In this study, we found that the transcriptional factor PrqR (Slr0895) in *Synechocystis* can perceive signals from ROS generated after shifting from prolonged darkness with glucose into high-light. The deletion mutant (DprqR) showed increased growth rate and decreased ROS content, whereas the complementary strain (CprqR) restored the growth characteristics, phenotypes and ROS status of WT, thereby establishing PrqR as a negative regulator of ROS.LC/GC-MS-based metabolic profiling also showed active ROS mitigation in DprqR mutant. Further study by qRT-PCR, ChIP-PCR and deletion of both *prqR* and *prqA* (DprqR-DprqA mutant) revealed that PrqR exerts this negative regulation of ROS removal by controlling the expression of *sodB* and *prqA* (*slr0896*). Furthermore, PrqR also found to control glucose metabolism by regulating a positive regulator of glucose metabolism, *sigE*, and its regulons. Results suggest that PrqR was involved in perceiving signals from ROS under physiological condition, as well as in regulating stress removal and glucose metabolism.

Aquatic environments with regular light dark cycle are typical for cyanobacteria. However, cyanobacteria are also living under adverse ecological conditions with prolonged darkness; for example, cyanobacteria can be found self-shadowing in dense planktonic and benthic communities, lake sedimentation, and soil water or dense aquatic accumulation produced at surface^1^,^2^. Thus, cyanobacteria could be a good model to understand metabolism and signaling network in adverse condition. In addition, as an evolutionary ancestor of plant photosynthetic machineries, cyanobacteria can also be a model platform to understand metabolism under similar prolonged dark conditions, which occurs in non-green organs of plants, such as roots, stems, and flowers^3^. Cyanobacteria respond to adverse conditions by generating reactive oxygen species (ROS) that can modulate transcription of genes in a genetic network and connect an appropriate riposte to the conditions^4^,^5^. But, how do cyanobacteria and plants perceive signals from ROS, coordinate signals from different sources, and reprogram metabolism under adverse physiological conditions is largely unknown.

In photosynthetic organisms, glucose can cause an accelerated ROS production^6^,^7^,^8^ or mitigate by generating NADPH by using the OPP pathway^6^,^9^. The addition of excess sugar can force plant cells into auto-heterotrophic condition, thereby suppressing photosynthesis and the Calvin cycle^6^. Under such conditions, NADPH production diminishes, eventually favoring ROS production^6^,^10^,^11^. To avoid this situation, signals exerted by light, sugar, and/or oxidative stress must be connected to balance sugar metabolism and ROS generation, as well as photosynthetic activity^12^,^13^. Signaling network of this kind has been started to be revealed. In plants, stress signals have been found interacting with glucose signaling through the hexokinase independent pathway^6^. For instance, the *psbA* gene, which encodes the D1 protein of photosystem II, converges signals from light, ROS and sugar^6^. By contrast, cytosolic glucose-6-phosphate dehydrogenase (G6PDH) of the OPP pathway mediates signals from both light and sugar^14^. Moreover, genes involved in ROS defense in cyanobacteria, such as chalcone synthase (CHS), glutathione-S-transferase (GST), and biosynthetic genes of ascorbate and carotenoid are also responding signals from sugar and light^6^,^15^,^16^,^17^,^18^. For example, genes of the carotenoid biosynthetic pathway, such as phytoene synthase (*crtB*), phytoene desaturase (*crtP*), ζ-carotene desaturase (*crtQ*), and β-carotene hydroxylase (*crtR*), were upregulated under high light, which is the ROS generating condition, but downregulated in the dark^19^. The expression of these genes can also be restored in the dark by adding glucose suggesting that the genes can perceive signal from ROS and glucose^20^. Similarly, *sigE*, a type 2 sigma factor with its positive role in glucose metabolism^21^,^22^, can respond to ROS^23^. However, previous studies are lacked to find appropriate experimental conditions that can provide platform to understand the genetic network in ROS mitigation and its relation with glucose metabolism under stress condition.

The model cyanobacterium, *Synechocystis* sp. PCC 6803 (hereafter *Synechocystis*) contains transcriptional factor that sense signals of stress from the environment and transduce the signals to coordinate the gene expression accordingly^24^. In *Synechocystis*, the mutant (*prq20*) of the transcriptional factor PrqR was previously shown to be adaptive resistance to an externally added methyl viologen (Paraquat)^25^. Additionally, the photosynthetic apparatus was found less affected in the *prq20* mutant than in the wild type (WT), as indicated by the damage generated by two strong-oxidative-stress producing compounds, namely, methyl viologen and benzyl viologen^26^. PrqR was found to contain a cis-acting regulatory sequence for auto-repression, and the frameshift mutation C134fs in the C-terminus DNA binding domain of PrqR inhibited this autoregulation^25^. Nevertheless, no information is available on the putative PrqR regulatory network under stress generating conditions.

With an initial goal to discover a response regulator that coordinate signals from both glucose and ROS, we established a unique nutritional condition, that is, GLC(+)DARK-LIGHT. In this condition, *Synechocystis* cultures were grown autotrophically (under illumination of 25 μmol m^−2^ s^−2^) until they reach exponential phase (OD_730_** **~ 0.40–0.45) in BG-11 media, with the addition of 5 mM glucose, samples were placed in the darkness for 72 h for GLC(+)DARK growth and then shifted back to grow under high light (80 μmol m^−2^ s^−2^) [GLC(+)DARK-LIGHT]. The results showed that oxidative stress was generated under GLC(+)DARK-LIGHT condition, and PrqR can be involved in perceiving ROS signals by repressing its downstream *prqA* gene, which encodes a putative multidrug-resistance protein. Furthermore, PrqR negatively regulated glucose metabolism by controlling the regulator of glucose metabolism. Overall, this study revealed that *prqR* plays a negative role in both glucose metabolism and ROS mitigation in *Synechocystis* under stress conditions.

## Results

### Phenotypic analysis of *Synechocystis* wild type and *prqR* mutants

**Figure 1:**
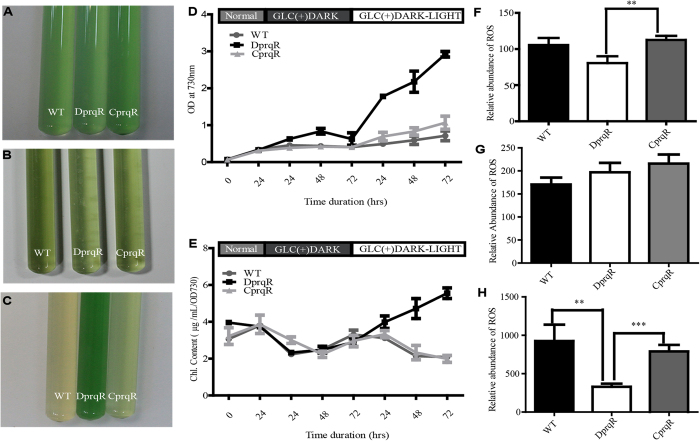
ROS generation and its effect under physiological condition and negative role of *prqR* in ROS mitigation in *Synechocystis*. Culture of WT, DprqR and CprqR under normal (**A**); 72 h under GLC(+)DARK (**B**); and 48 h under GLC(+)DARK-LIGHT (**C**) conditions; (**D**), growth characteristics of WT, DprqR and CprqR under normal, GLC(+)DARK, and GLC(+)DARK-LIGHT cultural conditions in different durations; (**E**), Chlorophyll content of WT, DprqR and CprqR under normal, GLC(+)DARK, and GLC(+)DARK-LIGHT cultural conditions in different durations; total ROS content of WT, DprqR and CprqR corresponding at normal growth condition before adding glucose (**F**); 72 h under GLC(+)DARK (**G**); and 48 h under GLC(+)DARK-LIGHT (**H**) conditions. In (**D**,**E**), the number represent the incubation time in hours and N = 5 ± SEM. In (**F–H**), N = 5 ± SEM and “*” represents statistical significance as indicated by Student’s t-test with maximum p-value of P < 0.05.

A previous study found that the *Synechocystis* mutant Prq20 (L17Q in *prqR* transcription factor) was resistant to methyl viologen, a strong ROS-generating chemical^26^,^27^. In the current study, to further determine the roles of the *prqR* gene in ROS removal and glucose metabolism at the molecular level, a *prqR*-disrupted mutant (DprqR) was generated by inserting a kanamycin cassette under *cpcB* promoter into the *Synechocystis* (glucose-intolerant strain) chromosome. For complementation, a strain with *prqR* gene overexpressed under a *psbA2* promoter along with the streptomycin cassette was also created in the DprqR mutant (CprqR) (the vectors and strains generated in this study are presented in Supplementary Fig. S1A). DprqR and CprqR were completely segregated on BG-11 plate supplemented with kanamycin and kanamycin + streptomycin (up to 100 μg/mL), respectively. The segregation was confirmed by PCR (Supplementary Figs S1B and S1C). WT, DprqR and CprqR showed normal growth in BG-11 liquid media in the presence of light at 25 μmol m^−2^ s^−2^, with shaking at 130 rpm and temperature of 30 °C (Fig. 1A). To assess glucose-dependent growth, we added 5 mM *D*-glucose into the culture when its OD_730_ was around 0.4 and then placed the culture into complete darkness [GLC(+)DARK] with shaking for different durations. Through the growth courses, the DprqR mutant grew almost similarly to WT and CprqR under GLC(+)DARK ; with subsequent placement of the cultures into high light, GLC(+)DARK-LIGHT(at 80 μmol m^−2^ s^−2^) led to approximately twofold increased growth of the DprqR mutant, whereas WT and CprqR showed significantly decreased growth as well as decolorization (Fig. 1B–D). To determine whether the phenotype attributed to the osmotic stress exerted by *D*-glucose, we repeated the experiments with *D*-sorbitol, which can produce an osmotic pressure similar to that *D*-glucose. However, all these strains grew equally normal (Supplementary Fig. S2), suggesting that the phenotype was not ascribed to osmotic stress. To determine whether the cells are dying under GLC(+)DARK and GLC(+)DARK-LIGHT conditions, we monitored the percentages of dead cells at two time points: at third day under GLC(+)DARK (72 h) condition and first day (24 h) under GLC(+)DARK-LIGHT condition. However, the results showed no significant changes in terms of cell death rates between WT and DprqR (Supplementary Fig. S3). Notably, the growth and chlorophyll content were found remarkably different in between DprqR and WT (and CprqR) after shifting from GLC(+)DARK to the GLC(+)DARK-LIGHT conditions (Fig. 1D,E).

### ROS decreased in DprqR mutant

Assuming that a sudden change of metabolism from glucose-dependent [GLC(+)DARK] condition to both glucose- and light-dependent [GLC(+)DARK-LIGHT] conditions would generate ROS, we determined the total ROS contents at three time points: before placement under GLC(+)DARK condition (normal cells), after 72 h under GLC(+)DARK and after 48 h in GLC(+)DARK-LIGHT condition. The total ROS contents were measured using membrane-permeant fluorescence indicator 5-(and-6)-chloromethyl-2′, 7′-dichlorodihydro fluorescein diacetate, CM-H2DCFDA (Invitrogen, Life technology). The results showed that under normal and GLC(+)DARK conditions, the ROS contents of WT, DprqR and CprqR were at similar levels (Fig. 1F,G), although a slightly higher accumulation of ROS in CprqR under normal condition was found and may be ascribed to the stronger *psbA2* promoter. However, significantly (more than twofold) higher ROS contents in WT and CprqR were found than those in DprqR under GLC(+)DARK-LIGHT condition after 48 h (Fig. 1H). In addition, no significant ROS accumulation difference was detected in WT, CprqR and DprqR in similar dark-light regime in the absence of glucose, that is, GLC(−)DARK and GLC(−)DARK-LIGHT (Supplementary Fig. S4). This finding suggests that GLC(+)DARK-LIGHT can cause ROS accumulation in *Synechocystis* cells, whereas the deletion of the *prqR* gene affected ROS mitigation.

**Figure 2:**
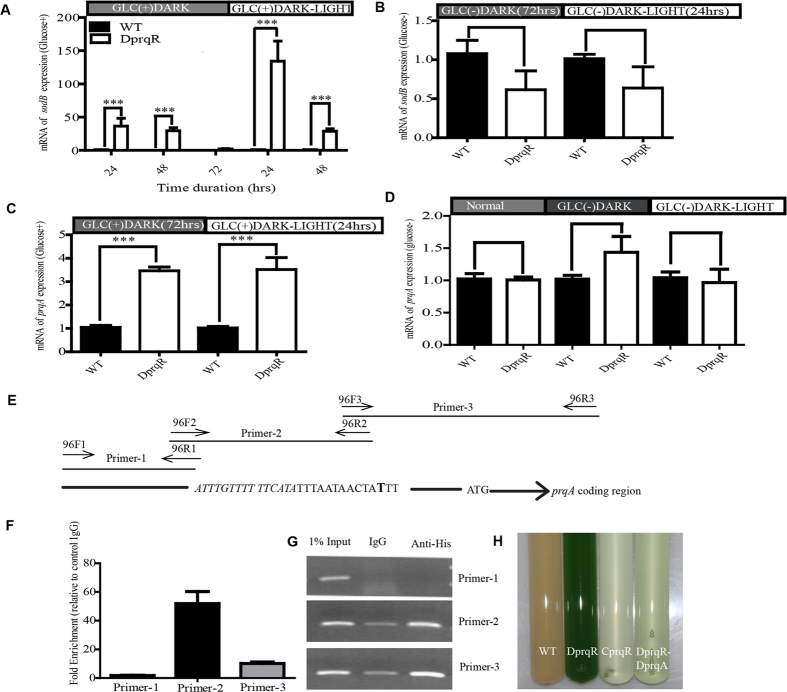
PrqR negatively regulates ROS-removing genes, *sodB* and *prqA*, in the presence of glucose. Expression of *sodB* at different time points of GLC(+)DARK and GLC(+)DARK-LIGHT (**A**); *sodB* expression in the absence of glucose, GLC(−)DARK (after 72 h) and GLC(−)DARK-LIGHT (after 24 h) (**B,C**), expression of *prqA* at GLC(+)DARK (after 72 h) and GLC(+)DARK-LIGHT (24 h); (**D**), expression of *prqA* in the absence of glucose, GLC(−)DARK (after 72 h) and GLC(−)DARK-LIGHT (after 24 h). In all the cases from (**A**–**D**), N = 5 ± SEM, and p-value P < 0.05, and were normalized by *rnpB* expression level. In (**E**), the designed primer sets (listed in Supplementary table S4) surrounded the predicted binding site of *prqA* (ATTTGTTTTTTCATA) promoter. ATG is the start codon of *prqA*; the bold “**T**” is the start site of transcription. The *prqR*-His was expressed in OEprqR strain. After ChIP, samples were assessed by quantitative PCR, and the relative enrichment was calculated against 1% input and IgG (**F**). For clarity, normal PCR with those primer sets were performed with 25 cycles (**G**). The picture in (**G**) was cropped from the gel run under similar condition in electrophoresis (400 V, 125 amps) with the sample from 25 cycle of PCR. The experiment was repeated twice and found similar results. The deletion of *prqR* and *prqA* from WT (DprqR-DprqA) restored the phenotype of WT and CprqR (**H**). The culture was taken in after 48 h under GLC(+)DARK-LIGHT condition.

Cyanobacteria exhibit diverse defense systems against oxidative stress, such as superoxide dismutase, which can remove superoxide radicals^4^. *Synechocystis* encompasses only one type of dismutase, that is, iron superoxide dismutase (FeSOD). Considering the role of FeSOD in ROS removal, we determined the expression order of *sodB* by qRT-PCR. The results showed that the *sodB* expression was upregulated in DprqR compared with WT at 24 and 48 h but equalized at 72 h under GLC(+)DARK condition. Subsequent shifting of the culture under GLC(+)DARK-LIGHT condition caused about 100-fold increase of *sodB* in DprqR strain compared with WT (Fig. 2A). To ensure that this fluctuation of the *sodB* expression in DprqR was ascribed to glucose rather than only high light, we maintained the GLC(−)DARK and GLC(−)DARK-LIGHT conditions but no significant upregulation or downregulation was observed (Fig. 2B).

### PrqR binds to the promoter of *prqA* and negatively regulates the expression of *prqA*

In the *Synechocystis* chromosome, *prqA* located downstream of *prqR* encodes a hypothetical protein belonging to the multidrug resistance protein of the MATE family. Analysis of gene expression showed that *prqA* was upregulated in DprqR by threefold and fourfold than the WT under GLC(+)DARK and GLC(+)DARK-LIGHT conditions, respectively (Fig. 2C). However, these differential expressions was not obtained in the absence of glucose (Fig. 2D). To determine whether *prqA* was the regulatory target of PrqR, we overexpressed *prqR* with a His-tag at its C-terminus under *cpcB* promoter in the WT. The expression of *prqR*-6xHis was confirmed by Western blot (Supplementary Fig. S1E). Chromatin Immunoprecipitation (ChIP) was conducted in accordance with the manufacturer’s manual (Beyotime, China). Before primers were designed for PCR, the 1000 bp upstream of the start codon of *prqA* was subjected to analysis with BDGP online software and BPROM^28^ for the transcription start site and transcription factor binding sites (Fig. 2E). Primers were then designed spanning the predicted binding site for an amplicon of 150 bp to 200 bp. After ChIP, DNA was used for PCR and the relative enrichment of DNA was analyzed against 1% input and IgG. The results showed that the putative *prqA* promoter was able to enrich approximately 50 and 10 fold of DNA, relative to control IgG, by primers 2 and primer 3, correspondingly (Fig. 2F). Furthermore, semi-quantitative PCR also found that primers 2 and 3 were able to amplify more DNA in the sample with anti-His antibody compared with the IgG control (Fig. 2G). This finding demonstrates that PrqR can bind directly with the promoter of *prqA*, which is immediately downstream of *prqR*. Similar analysis was performed on *gap2, sigE* and *sodB* genes (data are not shown). However, no significant binding was observed, suggesting that these genes were not regulated directly by PrqR.

We further deleted both *prqR* and *prqA* genes, resulting in the DprqR-DprqA mutant (Supplementary Fig. S1D). The DprqR-DprqA mutant exhibited similar growth of WT and DprqR under normal condition but we found significant growth retardation under GLC(+)DARK-LIGHT condition (Fig. 2H), similar to that observed for WT. Thus, *prqA* may be negatively controlled by PrqR, which can be essential for the survival under high ROS conditions, such as GLC(+)DARK-LIGHT.

### Glucose uptake and glycogen content varied in DprqR mutant

**Figure 3:**
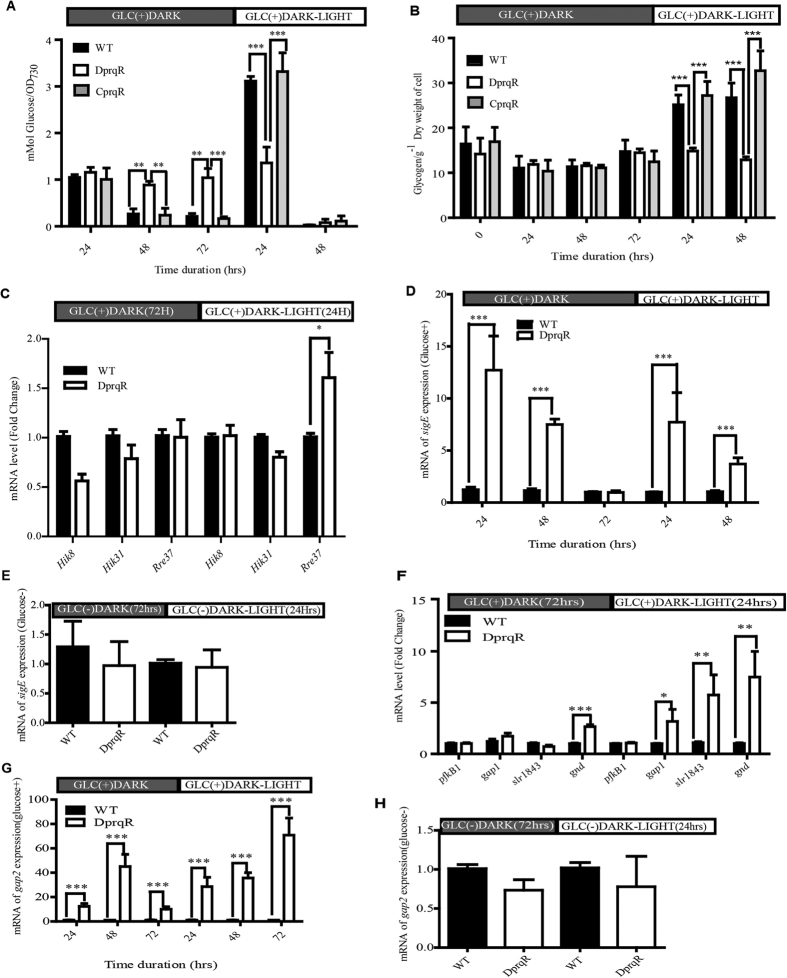
PrqR negatively regulates glucose metabolism by controlling the expression of individual and regulator genes. (**A**) Glucose uptake ability of WT, DprqR and CprqR under GLC(+)DARK and GLC(+)DARK-LIGHT conditions; (**B**) Glycogen content of WT, DprqR and CprqR under GLC(+)DARK and GLC(+)DARK-LIGHT conditions. The expression levels of three common regulators, *hik8* (*sll0750*), *rre37 (sll1330*) and *hik31 (sll0790*), of glucose metabolism under GLC(+)DARK (after 72 h) and GLC(+)DARK-LIGHT (after 24 h) conditions (**C,D**), represents the expression of *sigE* in different durations under GLC(+)DARK and GLC(+)DARK-LIGHT conditions, the number represents duration in hours; (**E**) is the expression of *sigE* in the absence of glucose, GLC(−)DARK and GLC(−)DARK-LIGHT conditions; (**F**) the expression level of regulons of *sigE*, two genes each in the glycolytic pathway (*pfkB1* and *gap1*) and the OPP pathway (*slr1843* and *gnd*) at GLC(+)DARK (after 72 h) and GLC(+)DARK-LIGHT (after 24 h) conditions; (**G**) *gap2* expression in different durations under GLC(+)DARK and GLC(+)DARK-LIGHT conditions; and (**H**) expression of *gap2* in the absence of glucose, GLC(−)DARK and GLC(−)DARK-LIGHT conditions. In (**A,B**), N = 3 ± SEM with three technical replications. “*” represents statistical significance, as shown by Student’s t-test with maximum *p*-value of < 0.05. From (**C**–**H**), N = 5 ± SEM, with maximum *p*-value < 0.05.

The mutant DprqR showed remarkable difference from WT and CprqR in both glucose uptake and glycogen content in the entire course of GLC(+)DARK and subsequent GLC(+)DARK-LIGHT condition. The glucose uptake in the first 24 h was found similar for WT, CprqR and DprqR mutants but declined more in WT and CprqR than in DprqR under GLC(+)DARK condition. After shifting from GLC(+)DARK to GLC(+)DARK-LIGHT both WT and CprqR showed a sharp increase in glucose uptake compared with DprqR (Fig. 3A) and after 48 h, DprqR consumed all the glucose added in the media, whereas WT stopped up-taking despite glucose remained in the media (Supplementary Fig. S5). This result suggests that DprqR continues glucose catabolism under the GLC(+)DARK condition, and more actively under GLC(+)DARK-LIGHT condition, until all the glucose is consumed. Glycogen aggregation was found to follow similar trends in the first 48 h under GLC(+)DARK but increased within 72 h in the WT, CprqR and DprqR mutants (Fig. 3B). The succeeding shift into the GLC(+)DARK-LIGHT condition led to a decreased glycogen content in the DprqR mutant, whereas the WT and CprqR maintained the same amount as those under the GLC(+)DARK condition.

### Dynamic expression of glucose metabolism regulators in DprqR mutant under GLC(+)DARK and GLC(+)DARK-LIGHT conditions

*D*-sorbitol did not affect cell growth under the D-SORBITOL(+)DARK-LIGHT condition (Supplementary Fig. S2). The possibility of osmotic stress was excluded. In addition, we washed the WT cells after 24 h under GLC(+)DARK-LIGHT. Afterward, we placed the cells into fresh media without glucose and found the cells resumed growth normally. These results led us to investigate the differential glucose metabolism abnormalities between WT and DprqR. Hence, we first targeted the following known regulators of the glucose metabolic pathway in *Synechocystis* through real-time qRT-PCR: a two-response regulatory protein Rre37 (*sll1330*) with HTH domain, which was previously found to control the expression of genes in glucose metabolic pathways in the presence of light and glucose^29^; Hik31 (*sll0790*) with regulatory role in glucose metabolism under heterotrophic growth^30^; a histidine kinase Hik8 (*sll0750*) involved in circadian rhythm and a regulator of glucose metabolism^31^; and a group 2 sigma factor SigE, which regulates the expression of several genes in glucose metabolic pathways under normal growth condition^21^,^22^. The results showed no significant difference among the expression of *hik8, hik31* and *rre37* between WT and DprqR under GLC(+)DARK condition (Fig. 3C). Under GLC(+)DARK-LIGHT condition, *hik8* and *hik31* exhibited a similar expression pattern in WT and DprqR, whereas *rre37* displayed a relatively higher expression in DprqR than that in WT (Fig. 3C); this finding is consistent with a previous report^29^. Moreover, *sigE* had been found with differential expression in DprqR compared with WT (Fig. 3D) under GLC(+)DARK (24 and 48 h) and GLC(+)DARK-LIGHT (24 and 48 h) conditions. Interestingly, the expression levels of *sigE* in WT and DprqR were similar at 72 h under GLC(+)DARK but differed after shifting into GLC(+)DARK-LIGHT (Fig. 3D); this finding suggest that the above time point is crucial for the expression of *sigE*. To determine whether the change is attributed to dark-to-high light or from glucose-dark [*i.e.*, GLC(+)DARK] to glucose-high light [*i.e.*, GLC(+)DARK-LIGHT], we incubated both WT and DprqR in GLC(−)DARK for 72 h and then shifted into GLC(−)DARK-LIGHT condition. RNA was isolated from the samples after 72 h in GLC(−)DARK and 24 h in GLC(−)DARK-LIGHT and real-time qRT-PCR was performed for analysis. The results showed no significant change of *sigE* expression in DprqR at the two time points (Fig. 3E); this finding suggests that glucose not high light alone caused the elevated *sigE* expression in DprqR.

In a previous study, the *sigE* mutant was found defective in glucose catabolism with decreased expression of phosphofructokinase, *pfkB1* (*sll1196*), glyceraldehyde-3-phosphate dehydrogenase (*gap1*) and pyruvate kinase (*pyk1*)^21^. To investigate role of *sigE* in terms of regulating genes of glucose metabolism under *prqR*-deficient background, we investigated the expression of three genes in the glycolytic pathway via real-time qRT-PCR, including two target genes of *sigE* (*i.e., pfkB1* and *gap1*). Although the results showed no significant change between WT and DprqR under either GLC(+)DARK or GLC(+)DARK-LIGHT conditions for two *sigE* target genes, except a slight increase of *gap1* expression in DprqR under GLC(+)DARK-LIGHT condition (Fig. 3F), the results showed upregulation of *gap2*, the gene encodes enzyme glyceraldyhyde-3-phosphate dehydrogenase in DprqR compared WT grown under GLC(+)DARK. In addition, we determined the expression pattern of *gap2* at different durations of GLC(+)DARK and GLC(+)DARK-LIGHT conditions (Fig. 3G). The results showed that the *gap2* expression in DprqR was more than 10 fold higher than that in WT throughout the GLC(+)DARK and GLC(+)DARK-LIGHT conditions. However, when we maintained a similar condition without glucose GLC(−)DARK for 72 h and then shifted to GLC(−)DARK-LIGHT for 24 h, no significant change of *gap2* expression between DprqR and WT was found (Fig. 3H). This finding suggests that glucose caused the remarkable change of *gap2* expression in D9prqR mutant under GLC(+)DARK and GLC(+)DARK-LIGHT conditions.

The OPP pathway is considered as the main route of carbon catabolism in *Synechocystis* and both *zwf* (*slr1843* encoded enzyme glucose-6-phosphate-1 dehydrogenase, G6PDH) and *gnd* (*sll0329* encoded enzyme 6-phosphogluconate dehydrogenase, 6PGD) play important roles in controlling the carbon flow regulated by *sigE*^21^,^22^. RT-PCR analysis showed that both *zwf* and *gnd* were slightly upregulated in DprqR under GLC(+)DARK condition compared with those in WT; a significant increase of *zwf* expression in DprqR was also observed under GLC(+)DARK-LIGHT condition (Fig. 3F), and this finding was consistent with the increased glucose and glycogen metabolism immediately after shifting from GLC(+)DARK to GLC(+)DARK-LIGHT.

### Metabolite abundance of central metabolic pathways in WT and DprqR mutant under GLC(+)DARK and GLC(+)DARK-LIGHT conditions

**Figure 4:**
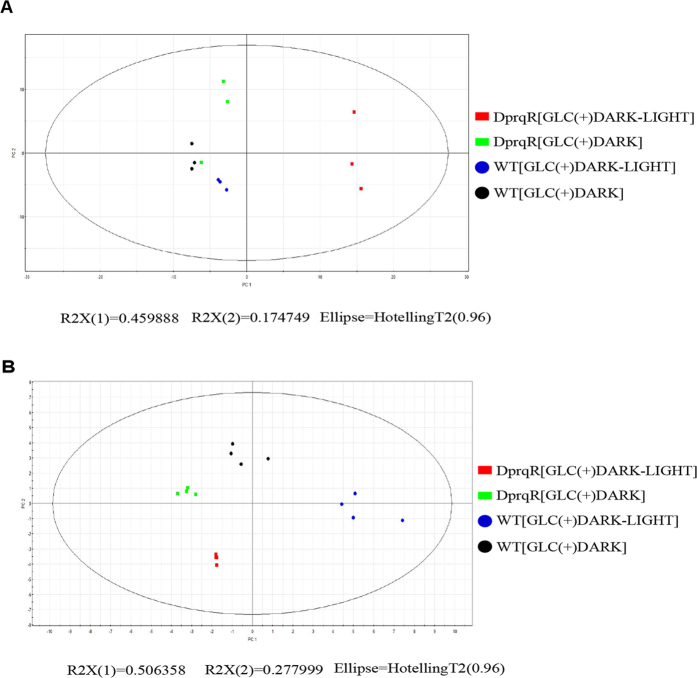
PCA analysis of targeted and non-targeted metabolites under two different conditions GLC(+)DARK (72 h) and GLC(+)DARK-LIGHT (24 h). (**A**) PCA analysis using SIMCA 12.0 software of non-targeted metabolites identified by GC-TOF-MS and (**B**) targeted metabolites detected by LC-MS. PC1 and PC2 referred as scores in principal components 1 and 2, respectively.

Metabolites were analyzed by both LC-MS and GC-MS in accordance with previously established protocols^32^,^33^. LC-MS-based analysis detected 23 targeted metabolites, mostly in glucose metabolic pathways, pyrimidine nucleotides, amino acid biosynthesis and energy-containing adenylated phosphate. GC-MS-based non-targeted metabolomics identified around 600 peaks, among which 183 compounds were selected after reproducibility analysis through repeated experiments. The abundance of each metabolite at GLC(+)DARK (72 h in dark) and GLC(+)DARK-LIGHT (24 h) was compared between DprqR and WT, and the significance of the changes between WT and DprqR was statistically evaluated by t-test (*p* < 0.05) (Supplementary Table S1 and S2). DprqR was significantly different from WT, thereby suggesting different metabolite profiles between GLC(+)DARK and GLC(+)DARK-LIGHT conditions. In addition, PCA analyses based on 23 metabolites detected by LC-MS (Fig. 4A) and 183 metabolites detected by GC-MS (Fig. 4B) showed a distinct separation between WT and DprqR under both GLC(+)DARK and GLC(+)DARK-LIGHT, suggesting that the deletion of *prqR* reprogrammed cellular metabolism significantly.

**Figure 5:**
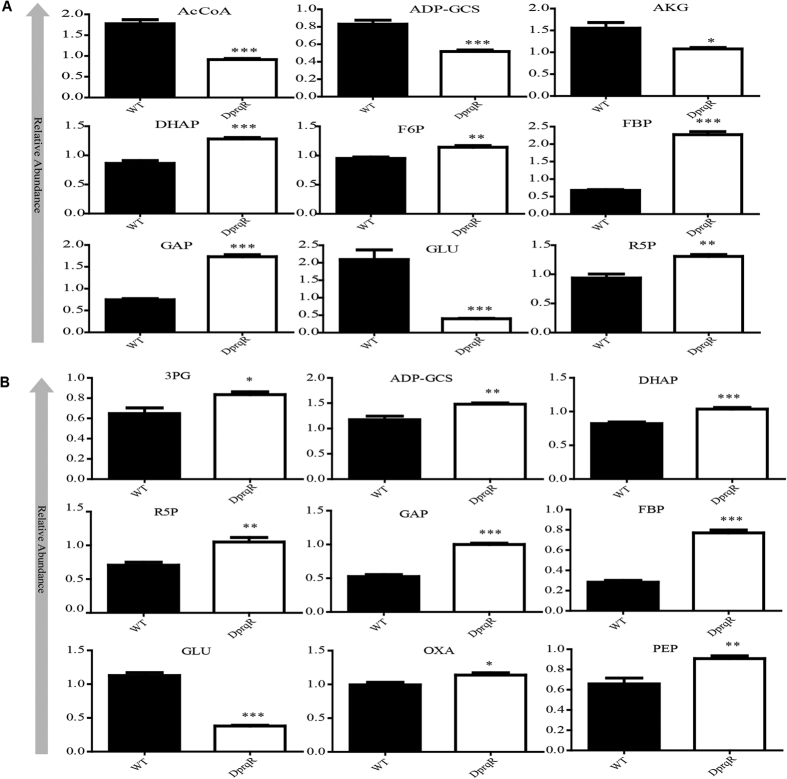
Comparison of metabolites identified. Statistical comparison of metabolites detected by LC-MS under GLC(+)DARK (after 72 h) (**A**) and GLC(+)DARK-LIGHT (**B**) condition (after 24 h). F6P, fructose-6-phosphate; FBP, fructose-1,6-bisphosphte; DHP, dihydroxyacetone phosphate; GAP, glyceraldehyde-3-phosphate; 3PG, 3-phosphoglycerate; PEP, phosphoenolpyruvate; AcCoA, acetyl-CoA; ADP-GCS, ADP-glucose; R5P, Ribose-5-phosphate; AKG, alpha-ketoglutarate; OXA, oxaloacetate; and Glu, glutamate. The star (*) indicates the level of significance, as shown by Student t-tests. The *p*-value of < 0.05 is considered significant with four independent replications. The error bar represents ± SEM.

**Figure 6:**
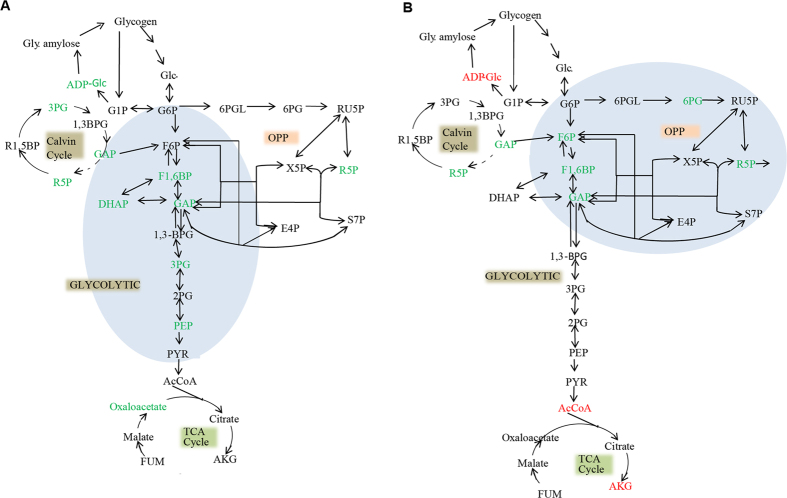
Change of metabolites in pathway. (**A**) under GLC(+)DARK condition and (**B**) under GLC(+)'DARK-LIGHT condition. “Green” represents increased abundance of the metabolites in DprqR compared with WT, “red” for decreased abundance and “black” was not significantly changed. The shadow is the pathway found to follow in particular conditions.

Significant differences between WT and DprqR were observed for metabolites of the glycolytic pathway, OPP pathway, and TCA cycle under GLC(+)DARK condition (Fig. 5A). For the glycolytic pathway, fructose-1,6-bisphosphate (FBP), glyceraldehyde-3-phosphate (GAP), 3-phosphoglycerate (3PG), and phosphoenolpyruvate (PEP), significantly increased in DprqR under GLC(+)DARK condition. Metabolites of gluconeogenesis (ADP-glucose), Calvin cycle [glyceraldehyde-3-phosphate (GAP), ribose-5-phosphate (R5P), and 3-phosphate glycerate (3PG)], TCA cycle (oxaloacetate) and oxidative pentose phosphate pathways [ribulose-5-phosphate (R5P)] were also found upregulated in DprqR compared with those in WT under GLC(+)DARK condition. The abundant changes of individual metabolites aligned in the metabolic pathway showed a sequential increase intermediate of the glycolytic pathway in DprqR, suggesting more glucose catabolism in GLC(+)DARK (Fig. 6A). Upon shifting from GLC(+)DARK to GLC(+)DARK-LIGHT, intermediates of the OPP pathway (R5P) (Figs 5B and 6B), as well as *D*-gluconic acid, a derivatization degradation product of 6-phosphogluconate, increased by twofold in DprqR (Supplementary Table S1); this finding suggests the active role of the OPP pathway in DprqR under the above mentioned condition. By contrast, the intermediates of the TCA cycle, namely, alpha-ketoglutarate and acetyl-CoA, decreased in DprqR.

### Changes of metabolites involved in oxidative stress mitigation

The metabolomics under oxidative stress in *Synechocystis* are not so available till now. Given that Cyanobacteria is evolutionary ancestor of plant and they share similar environmental habitat, we compared changed of metabolites in our work with some well-studied metabolic profile of plant under stress. Among many of such studies, *Noctor et al.*^34^ recently summarized a list of metabolites detected by GC/LC-MS that is more stably considered as marker to trace ROS status in *Arabidopsis* and Rice. The role and mechanism of many of these metabolites such as gluthathione, NADPH etc. in ROS acclimation are well-established. We compared the change of metabolites in our work with this information and listed. We found that under GLC(+)DARK condition, 2-monopalmitin, cadaverine, and *L*-rhamnose increased by more than twofold, whereas sorbitol and trehalose decreased more than twofold in DprqR than in WT (Supplementary Table S2). The most distinct change of metabolites involved in oxidative stress removal was observed under GLC(+)DARK-LIGHT condition. Myo-inositol, a sugar alcohol and signaling compound, decreased by approximately twofold in DprqR under GLC(+)DARK-LIGHT condition. Sugars such as lactose, maltose, *L*-sorbose and *D*-ribose were found to accumulate more than 1.5 fold in GLC(+)DARK-LIGHT condition in DprqR than in WT (Supplementary Table S1).

Glutathione (GSH) is considered as the most important defense metabolite against ROS damage in cells. GSH scavenges ROS and oxidize them to form glutathione disulfide (GSSG) by combining two GSH molecules with a sulfide bond. We were able to detect GSH by GC-TOF-MS, and the results showed that GSH was 1.75 fold higher in DprqR than in WT under GLC(+)DARK-LIGHT condition; however, no such difference was detected under GLC(+)DARK condition (Supplementary Table S1), and this finding was consistent with a previous report indicating that under the condition of oxidative stress, GSH declined and the redox state become more oxidized, thereby disrupting the cellular metabolic system^35^.

**Figure 7:**
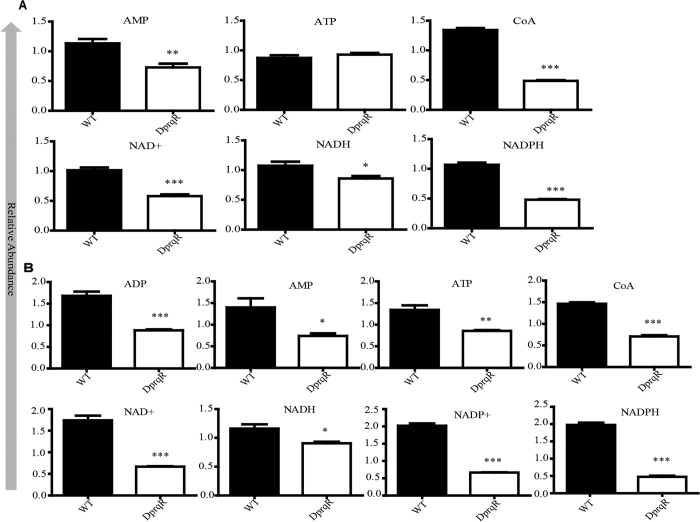
Comparison of abundance of pyridine nucleotide between WT and DprqR. Series (**A**) represents the relative abundance of pyridine nucleotide detected by LC-MS under GLC(+)DARK (after 72 h) and (**B**) under GLC(+)DARK-LIGHT condition (24 h). The data were developed by t-test of means from four independent replications. The star (*) indicates the level of significance by Student t-tests. The *p*-value < 0.05 is considered significant with four independent replications. The error bar represents ± SEM.

LC-MS based metabolite analyses revealed differential abundance of pyridine nucleotide and co-factors. Under GLC(+)DARK condition, ATP, AMP, NAD^+^, NADH, NADPH and CoA significantly decreased in DprqR mutant compared with those in WT. By contrast, ADP and NADP^+^ were remained unchanged (Fig. 7A). An almost similar trend was followed under GLC(+)DARK-LIGHT condition, in which ATP, AMP, NAD^+^, NADH, NADPH, Co-A, ADP and NADP^+^ decreased in DprqR mutant, compared with those in WT (Fig. 7B). The NADPH: NADP^+^ ratio decreased by ~42% and ~26% when shifted from GLC(+)DARK to GLC(+)DARK-LIGHT condition for WT and DprqR, respectively (Supplementary Table S5). Although the ratios of ATP:NADPH in WT and DprqR were similar under GLC(+)DARK condition, the ratio was relatively higher (1.90 unit) in the DprqR mutant than in WT under GLC(+)DARK-LIGHT condition (1.56 unit). This finding was consistent with a previous report indicating that changes in the ATP:NADPH ratio can lead to reponse in ROS generation^4^.

## Discussion

Studies have progressively elucidated that soluble sugars, such as glucose and sucrose, are strongly related to stress-induced ROS signaling in plants, cyanobacteria, *Saccharomyces*, and even mammals^7^,^9^,^36^,^37^. Unlike the known pro-oxidants and antioxidants which are only involved in ROS removal, soluble sugars such as glucose play a role in both ROS production and ROS-induced signal for scavenging. To clarify the mechanisms controlling ROS signaling pathways under environmental stress and their interplay with glucose metabolism, the focus should be shifted from studying signals under single stress to a combination of abiotic stresses in the presence of glucose to accurately represent the natural habitat. In contrast to previous studies, that focused on glucose metabolism under different trophic conditions, as well as cellular regulation of glucose uptake and metabolism^21^,^22^,^38^,^39^,^40^, the present study focused on GLC(+)DARK-LIGHT condition, which generates ROS (Fig. 1C,H), and provided a platform to investigate the complicated interaction of glucose and ROS as well as the signaling network.

**Figure 8:**
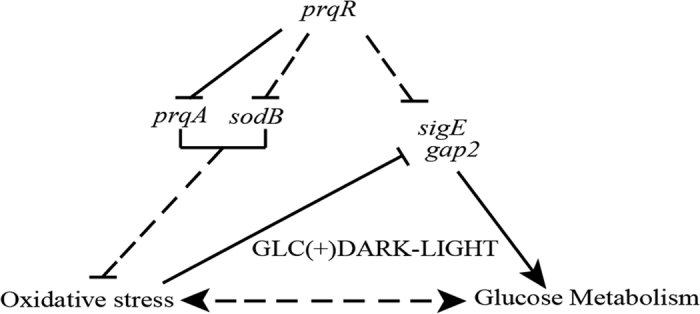
Network PrqR follows to control glucose metabolism and oxidative stress at GLC(+)DARK-LIGHT condition. PrqR negatively regulates *prqA* directly, and *sodB* indirectly; the two genes which expression is crucial for ROS removal from cells under GLC(+)DARK-LIGHT condition. The elevated oxidative stress (ROS) may have been suppressing glucose catabolism and downregulating *sigE* and *gap2* expression which together caused to develop the phenotype in WT and CprqR. The deleted *prqR* resulted upregulation of *prqA* as well as *sodB*, which helped to remove ROS. Thus, the cells with less ROS favored glucose metabolism through *sigE* and *gap2* upregulation, which eventually helped DprqR to grow faster in GLC(+)DARK-LIGHT condition. The “solid black” arrow represents the direct relation in contrast to the “interrupted” arrow is not. Overall, the *prqR* acts as a regulator and control oxidative stress and glucose metabolism in GLC(+)DARK-LIGHT condition.

Given that PrqR is a negative regulator of ROS^26^, the knockout mutant of *prqR* (DprqR mutant) differed from WT and CprqR for its lower ROS content was found with an increased glucose metabolism under GLC(+)DARK-LIGHT condition (Figs 1H and 3A,B, Supplementary Fig. S5); these findings reflect the ROS removal capacity is functional in DprqR under above-mentioned condition which can be related to glucose metabolism. In this study, we will discuss first how PrqR plays a role in ROS mitigation followed by glucose metabolism. PCA analysis of metabolites identified by LC-MS and GC-MS (Fig. 4A,B) showed reprogramming of metabolism in DprqR, whereas metabolic profiling revealed several key marker metabolite changes, which further indicated an increased ROS removal in DprqR. First, DprqR showed superiority of breaking down glucose following the glycolytic pathway under GLC(+)DARK condition (Figs 5A and 6A). This finding is in line with the ability of DprqR to enhance glucose uptake under GLC(+)DARK condition (Fig. 3A). In addition, major changes in shifting the glucose metabolism from the glycolytic pathway into the OPP pathway were observed under GLC(+)DARK-LIGHT condition (Fig. 6A,B). This switch is supposed to increase cellular NADPH given that the OPP pathway breaks down glucose to produce NADPH. On the contrary, we found decreased NADPH in DprqR cells under GLC(+)DARK-LIGHT condition (Fig. 7B). This finding explained that NADPH was used to reduce ROS-removing metabolites such as GSH. This NADPH-demand basis acceleration of the OPP pathway was also observed in photoheterotrophic condition in which NADPH production from photosynthesis was blocked and solely dependent on the OPP pathway^41^. Moreover, this re-routing of the central carbon metabolism, in favor of the OPP pathway to produce more NADPH, was previously reported in rice and yeast under oxidative stress removing cellular status^42^,^43^. *Takahashi et al.* found increased accumulation of glycolytic pathway intermediates such as fructose-6-phosphate, 3-phosphoglycerate, 2-phosphoglycerate, and phosphoenolpyruvate in mixotrophic condition in *Synechocystis*, similar to GLC(+)DARK-LIGHT in current study, except the prior extension of GLC(+)DARK incubation^38^. Overall, this study^38^ suggested an active mode of glycolytic pathway under mixotrophic condition which is sharply contrasting with our findings obtained under GLC(+)DARK-LIGHT condition. Thus, this particular information agrees with the understanding that glucose and ROS reprogrammed the central metabolic pathway favoring ROS removal in DprqR under GLC(+)DARK-LIGHT condition. Second, the reduced GSH increased in DprqR under GLC(+)DARK-LIGHT condition (Supplementary Table S1). This observation is similar to previous report indicating that the response of GSH to ROS acclimation is a time-series event and a successful acclimation accumulate reduced GSH with unsuccessful acclimation increased GSSG/GSH ratio^35^. Third, myo-inositol, a sugar alcohol which is a strong scavenger of ROS, was found with a sharply decreased concentration in DprqR mutant (Supplementary Table S1); this finding was consistent with a previous study in a *Arabidopsis cat2* mutant, in which the ROS scavenger metabolites decreased in concentration during stress acclimation^44^,^45^. Finally, the results showed that the TCA cycle was perturbed in cells under GLC(+)DARK-LIGHT condition (Fig. 6B), which is within the expectation as a number of enzymes, including aconitase and 2-oxogluterate, are known to be affected during acclimation of oxidative stress^34^,^46^. Overall, metabolomics analysis demonstrated that the decreased ROS content and active ROS removal mechanism in DprqR mutant cell under GLC(+)DARK-LIGHT condition confirmed the negative regulation of ROS by PrqR (Fig. 8).

These properties of the DprqR mutant led to the assumption that PrqR functions as a regulatory protein for ROS removal. Therefore, a search for possible targets of PrqR ended up establishing PrqR as a negative regulator of *prqA* and *sodB*; qRT-PCR showed an upsurge of the *prqA* and *sodB* expression in DprqR under both GLC(+)DARK and GLC(+)DARK-LIGHT conditions (Fig. 2A,C). Although very limited information is available about *sodB* regulation by sugars in cyanobacteria, the participation of glucose in antioxidant induction has been reported^20^. Studies suggested that sugar-regulated genes involved in anti-oxidative systems are under control of sugar sensors^14^, leading to a hypothesis that both *sodB* and *prqA* may be regulated directly by PrqR. In contrast to this initial expectation, the ChIP results showed that PrqR bound only to the *prqA* but not *sodB* promoter (Fig. 2F,G); thus the result suggests that PrqR regulates *prqA* directly and *sodB* indirectly. PrqA is a putative multidrug resistance efflux protein previously shown to respond to methyl viologen along with PrqR^47^. Phenotype of DprqR-DprqA mutant under GLC(+)DARK-LIGHT condition (Fig. 2H), which was similar to WT, suggesting that *prqA* expression in DprqR mutant was crucial in ROS mitigation. However, the exact mechanism of how does *prqA* expression mitigate ROS is yet to be known. Altogether, the lower ROS content and elevated expression of ROS removing genes in DprqR implies that PrqR is a negative regulator of ROS under GLC(+)DARK-LIGHT condition (Fig. 8).

Instead of feeding the OPP pathway by glucose and replenishing NADPH that helps to scavenge ROS in plants under stress^48^,^49^, glucose was observed to generate ROS and impaired its metabolism under GLC(+)DARK-LIGHT condition in WT and CprqR (Fig. 1C,H). The enhanced glucose metabolism is understandable with overexpression of *sigE*, which is the positive regulator of glucose metabolism in *Synechocystis*^21^ (Fig. 3D), and its regulon in OPP pathway (*i.e.*, *zwf* and *gnd*) (Fig. 3F)^21^,^50^. However, Chip-PCR measurement confirmed that PrqR does not regulate *sigE* directly (data not shown). Thus, an alternative explanation of glucose metabolism regulation in DprqR mutant through *sigE* expression under GLC(+)DARK-LIGHT condition implies that DprqR mutant mitigated ROS under GLC(+)DARK-LIGHT condition, thereby avoiding a negative consequence of ROS on *sigE* allowing *sigE* expression in the presence of glucose. Similarly, our result indicating that *gap2* is overexpressed in DprqR under GLC(+)DARK-LIGHT condition (Fig. 3G,H), can be fit with the suggestion relevent to a previous study^51^. Together, this suggestion justifyes our hypothesis that DprqR helps to remove stress from cells grown under high light at GLC(+)DARK-LIGHT condition (Fig. 8). However, this suggestion cannot explain the increased expression of *gap2* in DprqR at GLC(+)DARK condition, during which no significant accumulation of ROS occured.

In summary, the established GLC(+)DARK-LIGHT condition provides a platform to determine the ROS signaling and glucose metabolism in *Synechocystis*. Our results, as well as physiological analyses from a previous study^26^, demonstrated that the response regulator PrqR is involved in the regulation of glucose metabolism and oxidative stress acclimation in *Synechocystis* (Fig. 8).

## Materials and Methods

### Conditions of growth and maintenance for cyanobacteria

The *Synechocystis* sp. PCC 6803 strain used in this study was kindly provided by Prof. Weiwen Zhang (Tianjin University, China). *Synechocystis* was cultivated in BG-11 media (both solid and liquid)^52^. For normal growth, the culture was maintained in a shaker photo-incubator under continuous illumination at 25 μmol m^−2^ s^−2^, with shaking at 130 rpm and temperature of 30 °C. The mutant culture was supplemented with various antibiotics at different concentrations, as mentioned in the text (for DprqR mutant line, up to 100 μg/mL kanamycin; for CprqR, 100 μg/mL kanamycin + 100 mg/mL spectinomycin; for OEprqR, 100 μg/mL kanamycin; and for DprqR-DprqA, 100 μg/mL kanamycin). For heterotrophic [GLC(+)DARK] growth, 5 mM glucose was added into the culture which was then maintained in the dark by wrapping with aluminum foil while shaking at 130 rpm and temperature of 30 °C. In this study, we maintained a special condition called GLC(+)DARK-LIGHT in which cells were inoculated in liquid BG-11 media and kept for normal culture until the OD_730_ was around 0.4; we then added 5 mM glucose and placed the samples under GLC(+)DARK condition for 3 days with subsequent shifting into high light (at 80 μmol m^−2^ s^−2^) for 2 or 3 days. For plate culture, the solid BG-11 on plate was always maintained in photo-incubator with continuous illumination at 25 μmol m^−2^ s^−2^ and temperature of 30 °C. Antibiotics had been used during maintenance or selection of mutant strains but not when comparative study was conducted between WT and different mutants.

### Cloning strategies, transformation and generation of stable strains

The constructed vectors and strains developed by transforming those vectors are listed in Fig. S1A. The vectors were constructed by combining the normal restriction-digestion-ligation system and fusion system, as described by Zhang *et al*.^53^. The two vector’s backbones, namely, pUC19 and pBluescript KS (+), were used to construct all other vectors. The primers used in these constructions are listed in Supplementary Table S3. The constructed vectors transformed into *Synechocystis* following the method described by Liu *et al*.^54^. Colonies that appeared on the plate were streaked several times (at least 10 times) to obtain complete segregation. The mutant was confirmed by PCR, and positive clones were set in liquid culture supplemented with appropriate antibiotics.

In DprqR mutant (“D” stands for deletion), 245 bp out of 645 bp of *prqR* gene was deleted and a kanamycin cassette was inserted. The CprqR strain (“C” represents complementation) was generated by expressing the *prqR* gene under *psbA2* promoter and spectinomycin under *cpcB* promoter in DprqR mutant by inserting in a neutral site (NS) at *slr1311*. For ChIP assay, we attached a 6XHis Tag at the C-terminus of the *prqR* gene, which was expressed under *psbA2* promoter in WT strain at NS site of *slr1311*. To delete the two neighboring gene *prqR* and *prqA*, vector was designed using an upstream fragment of *prqR* and a downstream fragment of *prqA* flanked with kanamycin cassette under *cpcB* promoter.

### ChIP-PCR

Chip-PCR was conducted using all reagents and protocols provided by Beyotime (Shanghai, China). Briefly, 40 mL of cell culture was collected from the normal culture of OEprqR strain, crosslinked using 1% formaldehyde and stabilized using 125 mM (final concentration) glycine. Sonication was performed as follows: 1 s ON, 2 s OFF for 18 min with 25% amplitude, to achieve the DNA fragment length of 400 bp to 800 bp. After centrifugation, the supernatant was transferred (about 0.2 mL) into a 2 mL ice-bathed tube. ChIP was performed in accordance with the protocol given by Beyotime (Shanghai, China).

The real time q-PCR was set with a 25 μL system (12.5 μL of SYBR GreeN, 0.5 × 2 Primers, 1 μL of sample, 1 μL of 1% input, 9.5 μL of ddH2O). Three pairs of primers were used for each targeted set (Supplementary Table S4). The DNA sample eluted with normal unconjugated mouse IgG was used as control.

Semi-quantitative PCR was set using *Taq* 2X Master Mix Kit (New England Biolabs Inc). The reaction was set in a 25 μL system (12.5 *Taq* 2X Master Mix, 0.5 × 2 Primers, 1 μL each of sample and 1% input, and 10.5 ddH2O) for 25 cycles.

### GC/LC-MS based metabolic profiling

For GC-MS, approximately 3 × 10^9^ cells were collected from the culture under GLC(+)DARK and GLC(+)DARK-LIGHT conditions and placed into −80 °C for at least 2 min to stop further metabolic activity. Cells were collected by centrifugation, washed twice with ice-cold NaCl (0.9%) and placed in tubes in the presence of liquid nitrogen. Up to 1.5 mL of methanol-ddH_2_O (1:1 v/v) was added along with 10 μL of L-2-chlorophenylalanine (0.3 mg/mL in dH2O). Sonication was conducted as follows: 5 s ON, 10 s OFF for 15 min at 90% amplitude, and then repeated thrice. The supernatant was dried up completely by freeze drying with continuous flow of nitrogen. Samples were derivatized and run under GC-TOF-MS following the method reported by Liu *et al*.^32^. The data were acquired in full-scan mode with the m/z range of 30–550 at a rate of 20 spectra per second following a solvent delay of 360 s. The acquired data were processed and analyzed as previously described^32^,^55^. Metabolite identification was performed by Chroma TOF linked with spectral library databases of National Institute of Standards and Technology (NIST) (2005). Other original standards were used to further validate the data. The dataset extracted in MS files from GC/TOFMS analysis was exported into NetCDF format by ChromaTOF software (v3.30, Leco Co., CA). CDF files were processed using custom scripts (revised MATLAB toolbox, hierarchical multivariate curve resolution (H-MCR), developed by Jonsson *et al*.^56^,^57^ in MATLAB 7.0 (The Math Works). Principal component analysis (PCA) was performed with SIMCA-P_12.0 software package (Umetrics, Umeå, Sweden) and GraphPad Prism version 5.01.

For LC-MS analysis, samples with equal cells number were collected at two time points: 72 h under GLC(+)DARK and 24 h under GLC(+)DARK-LIGHT condition. The pellet was centrifuged and collected quickly, frozen in liquid nitrogen and then preserved it at −80 °C before further use. Metabolites were extracted by quenching following the protocol described by *Su et. al*.^33^. In quenching metabolites, the frozen pellet was dissolved in 500 μL of 100% LC-MS grade methanol and placed for thawing on dry ice. The supernatant was collected and stored at −80 °C. The LC-MS experiment was conducted using an Agilent 1260 series binary HPLC system (Agilent Technologies, Waldbronn, Germany) attached with an Agilent 6410 triple quadrupole mass analyzer equipped with an electronic spray ion (ESI) source. Aqueous 10 mM tributylamine (pH 4.95, adjusted with acetic acid) and 100% HPLC-grade methanol were used as Mobile Phase A (MPA) and Mobile Phase B (MPB), respectively. Agilent Mass Hunter workstation LC/QQQ acquisition software (version B.04.01) was applied for data processing and Agilent Mass Hunter workstation qualitative analysis software version (B.04.00) applied for statistical analysis. The data obtained were analyzed using SIMCA 12.0 software (for PCA analysis), MeV4_9_0 (for Heat Map), and GraphPad Prism version 5.01 (t-test and others).

### Growth and chlorophyll content measurements

The growth models of WT, DprqR and CprqR were studied under normal, GLC(+)DARK and GLC(+)DARK-LIGHT conditions by detecting the optical density (OD) at different wavelengths by using a spectrophotometer (METASH). OD_730_ was used to determine growth curve. OD_678_, OD_720_ and OD_750_ were also measured, and the total chlorophyll content was quantified using following formula; 14.94 (OD_678_–OD_750_)−0.616 (OD_720_–OD_750_), described by^58^.

### RNA isolation, cDNA synthesis, and qRT-PCR

Total RNA from 50 mL of culture had been isolated using TRIzol (Invitrogen) following the modified method described by *Schwarzkopf et al.*^59^. The collected RNA was then treated with DNase1 (RNase free), deactivated DNase1 to remove contaminated DNA following the provided protocol. Thus, the DNA-free RNA was subjected to convert cDNA applying kits, SuperScript^®^ III First-Strand Synthesis System (Invitrogen). qRT-PCR was performed by applying FastStart Universal SYBER Green (ROX) (Roche) reagent in Applied Biosystems 7500 RealTime PCR System. The RNA subunit of ribonuclease P subunit B (*rnpB*) served as an internal control. The primers used to quantify gene expression are listed in Supplementary Table S4.

### ROS measurement

We measured the total ROS by using membrane-permeant fluorescence indicator 5-(and-6)-chloromethyl-2′, 7′-dichlorodihydro fluorescein diacetate, CM-H2DCFDA (Invitrogen, Life technology), following the protocols described by *Hakkila, et al.*^23^ and provided by Life Technology. Samples from normal culture, GLC(+)DARK culture (72 h) and GLC(+)DARK-LIGHT (24 h) were taken to estimate the total ROS content. Up to 25 mM CM-H2DCFDA was added to 1 mL of culture in 2 mL tube and then mixed. About 1 mL of culture was also obtained without adding any CM-H2DCFDA and considered as control (auto-fluorescence). Samples were then incubated for 90 min. in complete dark at 32 °C. Cells were washed with BG-11 media and suspended with BG-11 media to reach the final volume of 0.5 mL. Up to 200 μL of cell suspension was pipetted to a white 96-well microtiter plate. Fluorescence from CM-H2DCFDA treated and untreated cell (auto-fluorescence) was measured using Synergy Multi-Mode Reader (BioTek). The data measured immediately after dark incubation were considered as “Initial Reading” and then incubated in the corresponding condition for 1 h and measured the fluorescence for “Final Reading.” The strategy was followed both for treated and untreated (auto-fluorescence) samples. The ROS content was then calculated following the equation, ROS content = (IR_T_-FR_T_)-(IR_UT_-FR_UT_) where IR_T_, Initial Reading of Treated sample; FR_T_, Final Reading of Treated sample; IR_UT_, Initial Reading of Un-treated sample; and FR_UT_, Final Reading of Un-treated sample.

The excitation and detection emission had been set to 485/20 nm and 535/20 nm respectively with sensitivity at 50, optic position in bottom and normal read speed. BioTek’s Gen5™ 1.10 Reader Control and Data Analysis Software were used to set and analyze the data.

### Glucose (extracellular) and glycogen measurements

Both glycogen and glucose were measured described elsewhere^60^. For glycogen quantification, 3 mL sample was obtained and centrifuged; the pellet was collected and washed with sterile ddH_2_O and then freeze dried. The weight of the dried cell was recorded. The cell was then suspended in 30% w/v KOH, mixed and incubated at 97 °C for 2 h. Ice-cold ethanol was added to the final concentration of 70% to 75% and incubated on ice for at least 2 h. The mixture was centrifuged, the supernatant was removed, and the pellet was washed with 98% ethanol. The glycogen pellet was dried at 60 °C for 10 min and then dissolved in 100 mM sodium acetate (pH 4.5). The dissolved glycogen was hydrolyzed enzymatically, by treating with 2 mg/mL amyloglucosidase (Sigma) at 60 °C for 2 h, into glucose. The glucose obtained from glycogen and extracellular (in media added externally) was measured using a Glucose (GO) Assay kit (Sigma-Aldrich) in accordance with the manufacture’s protocol. For glycogen measurement, data were normalized by cell dry weight. To understand the glucose uptake ability of the cell, kinetics of the decrease of the glucose content in the growth medium under GLC(+)DARK and GLC(+)DARK-LIGHT conditions were calculated. The data was normalized by OD_730_ according to time duration.

## Additional Information

**How to cite this article**: Khan, R. I. *et al*. Transcriptional regulator PrqR plays a negative role in glucose metabolism and oxidative stress acclimation in *Synechocystis* sp. PCC 6803. *Sci. Rep.* **6**, 32507; doi: 10.1038/srep32507 (2016).

## Supplementary Material

Supplementary Information
