# Supplementary material for: Transcriptional regulator PrqR plays a negative role in glucose metabolism and oxidative stress acclimation in Synechocystis sp. PCC 6803
Source: Sci Rep. 2016 Sep 1;6:32507. doi: 10.1038/srep32507 (PMC5007503; doi:10.1038/srep32507)
Supplement: Supplementary Information [file srep32507-s1.pdf]

Supplementary Information

Transcriptional regulator PrqR plays a negative role in glucose metabolism and oxidative stress acclimation in *Synechocystis* sp. PCC 6803

Md. Rezaul Islam Khan [1,2], Yushu Wang [1], Shajia Afrin [1], Bing Wang[2], Yumin Liu [3], Xiaoqing Zhang [4,5], Lei Chen [4,5], Weiwen Zhang [4,5], Lin He [1]*, and Gang Ma [1,2]*

[1] Bio-X-Renji Hospital Research Center, Renji Hospital, School of Medicine, Shanghai Jiao Tong University, Shanghai 200240, P.R. China;

[2] Bio-X Institutes, Key Laboratory for the Genetics of Developmental and Neuropsychiatric Disorders (Ministry of Education), Shanghai Jiao Tong University, Shanghai 200240, P.R. China;

[3]School of Electronics and Information Engineering, Tongji University, Shanghai 201804, China;

[4] Instrumental Analysis Center of Shanghai Jiao Tong University, Shanghai 200240, P.R. China;

[5] Laboratory of Synthetic Microbiology, School of Chemical Engineering & Technology, Tianjin University, Tianjin 300072, P.R. China.

[6] Collaborative Innovation Center of Chemical Science and Engineering, Tianjin, P.R. China.

* Corresponding authors

Gang Ma: magang@sjtu.edu.cn, Fax: 86-21-34207232

Lin He: helinhelin3@vip.163.com, Fax: 86-21-62932059

A Vector design & strain generation

Name of vectors	Corresponding strain	Selected Antibiotic
pUC95UCpcKana95D	DprqR (deletion of slr0895)	Kanamycin
pBL11UpsbA2slr0895His CpcSmr11D	CprqR (Complementation of DprqR)	Kanamycin + Spectinomycin
pBL11UpsbA2slr0895His CpcKana11D	OEprqR (overexpression of slr0895 in WT)	Kanamycin
pBL95UCpcKana96D	DprqR - DprqA(Deletion of slr0895 & slr0896 togather from WT)	Kanamycin

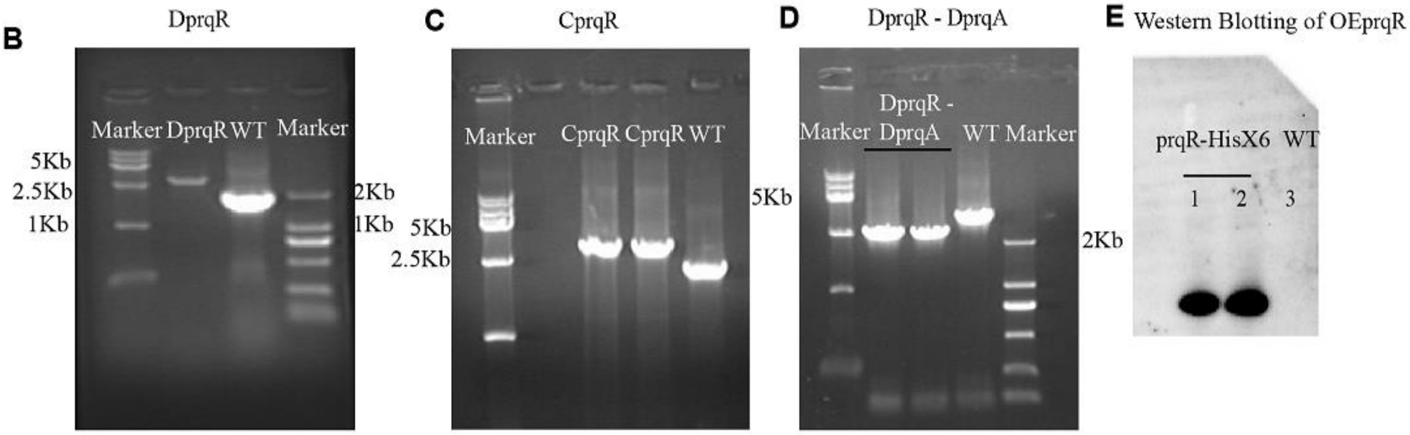

Supplementary Figure S1. List of vectors constructed and PCR Identification of mutant and transgenic strains. (A), List of name of vectors, the corresponding strain generated and the antibiotic used for selection. (B), PCR identification of DprqR strain using primer Xba1-D95UF and EcoRI-D95DR (listed in Supplementary Table S3). In DprqR, 243bp out of 564 bp of slr0895 (*prqR*) was designed to delete and kanamycin under *cpcB* promoter (total 1492bp) was inserted in place. The primer pair amplified 1962 bp from WT and 3211bp from DprqR strain. The extra size of DprqR is the sequence of *cpcB*-Kanamycin (1492 bp). (C), genotyping of CprqR strain using PCR. The *prqR*-HisX6 under *psbA2* promoter along with Streptomycin resistance gene (*smrR*, 792bp) under *cpcB* promoter was expressed. The primer pair Kpn1-1311UF and Spe1-1311DR was used to amplify 2432 bp from WT and 3333 bp from CprqR strain. The extra size in CprqR strain is sequence of psbA2-*prqR*-HisX6 and *cpcB*-*smrR*. (D), genotyping of DprqR-DprqR strain by PCR. The two genes, *prqR* and *prqR* are located side by side. Total 3217 bp was amplified using primer pair Xba195UF and Kpn196DR. A total 2540 bp was amplified from DprqR-DprqR strain that includes *cpc**B***-KanaR. (E), Western Blotting result of 6XHis attached *prqR* expression in OEprqR (column 1 and 2 represent two separate clones of OEprqR) strain. To do PCR, genomic DNA was isolated and PCR was set with Taq polymerase (Thermo Scientific).

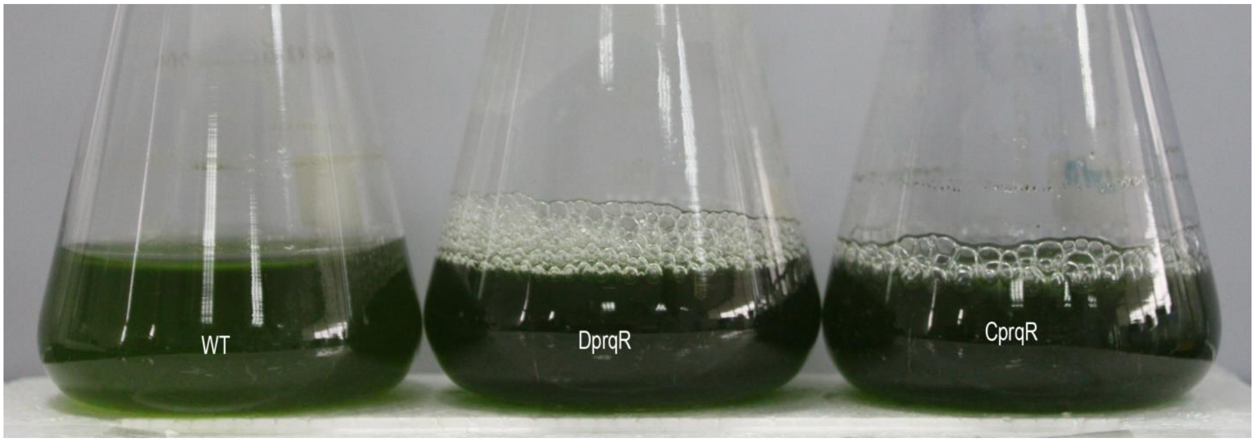

Supplementary Figure S2. Phenotypic analyses with D-sorbitol. The culture was maintained with D-sorbitol, instead of D-glucose, and incubated 72 h in the darkness [D-SORBITOL(+)DARK condition] and shifted into high light [D-SORBITOL(+)DARK-LIGHT condition). The picture was taken after 72 h in high light [D-SORBITOL(+)DARK-LIGHT].

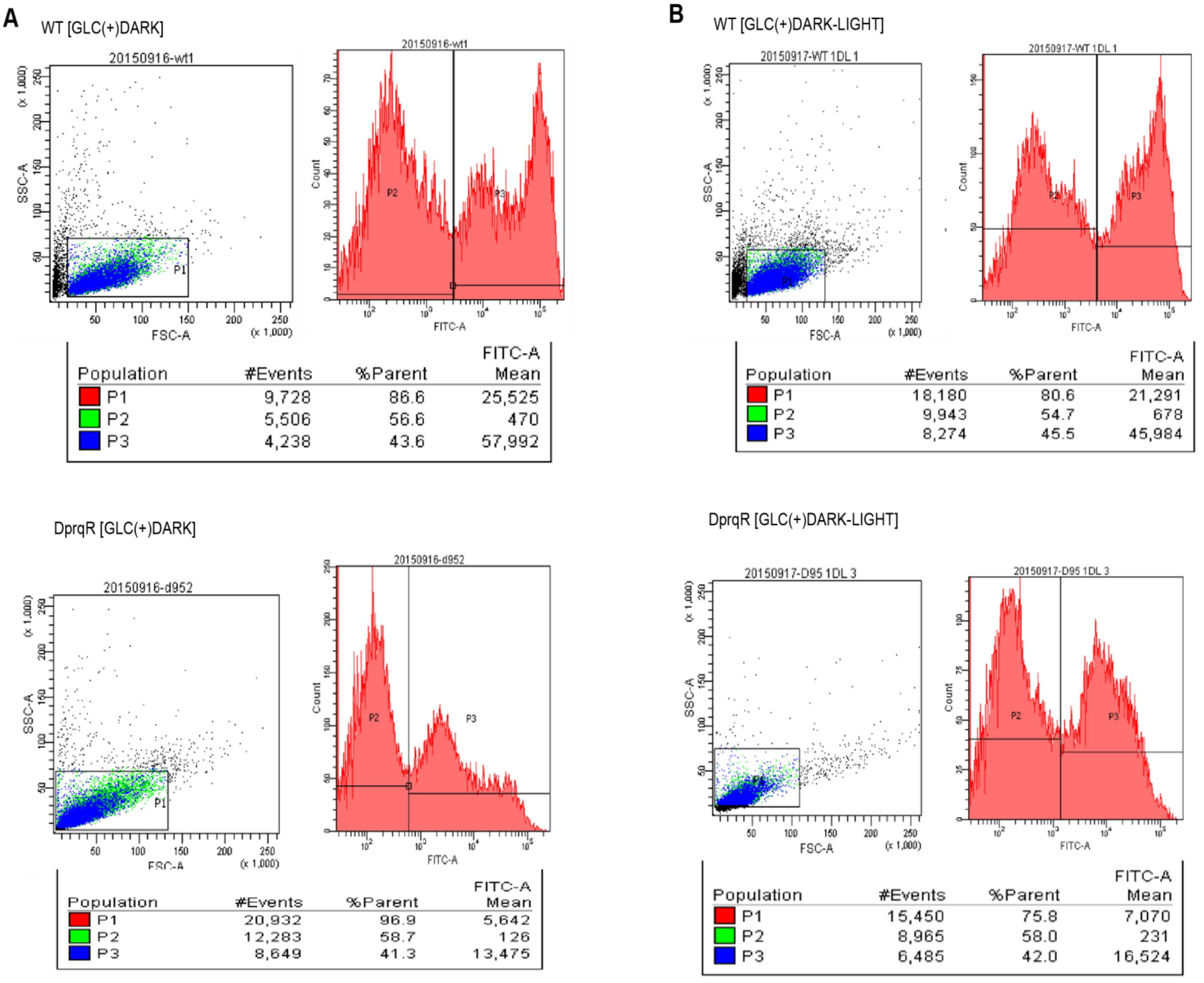

Population	#Events	%Parent	FITC-A Mean
P1	9,728	86.6	25,525
P2	5,506	56.6	470
P3	4,238	43.6	57,992

Population	#Events	%Parent	FITC-A Mean
P1	18,180	80.6	21,291
P2	9,943	54.7	678
P3	8,274	45.5	45,984

Population	#Events	%Parent	FITC-A Mean
P1	20,932	96.9	5,642
P2	12,283	58.7	126
P3	8,649	41.3	13,475

Population	#Events	%Parent	FITC-A Mean
P1	15,450	75.8	7,070
P2	8,965	58.0	231
P3	6,485	42.0	16,524

Supplementary Figure S3. FACS analysis of death cell percentage; (A), the row represent the percentage of death cell in WT and DprqR at GLC(+)DARK (after 72 h) condition; and (B) for GLC(+)DARK-LIGHT (24 h). P2 is live cell count while P3 is death cell. The cell was stained with SYTOX® Green Dead Cell Stain dye (invitrogen) and cells were counted following the given protocol using the BD FACSCalibur™ (BD Bioscience).

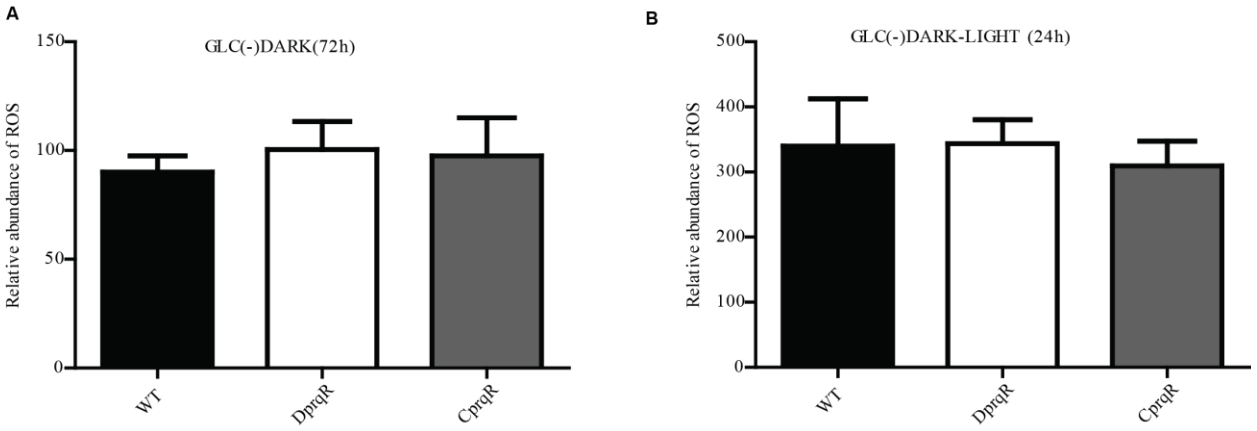

Supplementary Figure S4. ROS accumulation in the absence of glucose. (A), ROS was measured from WT, DprqR and CprqR in dark (72 h) in the absence of glucose [GLC (-)DARK]; and (B), in high light (24 h) shifted from dark in the absence of glucose [GLC(-)DARK-LIGHT]. Here, N=3±SEM.

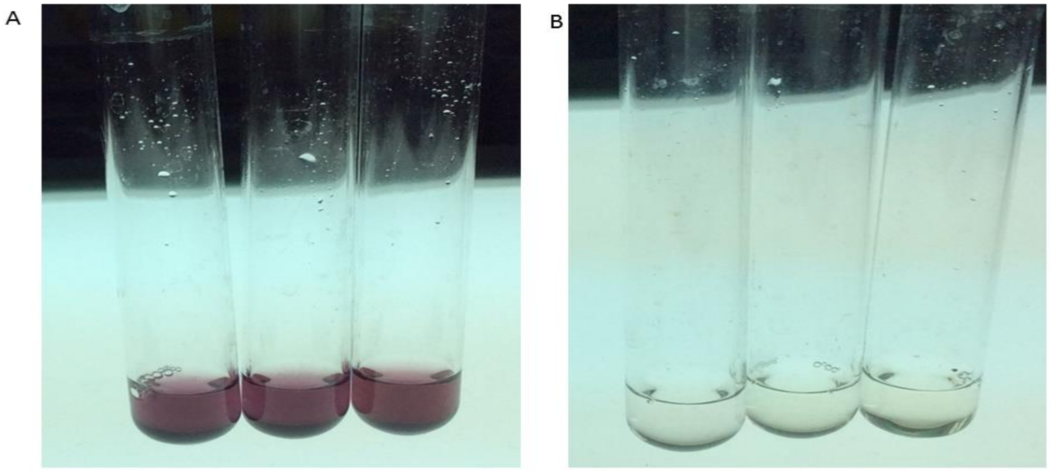

Supplementary Figure S 5. Presence of glucose in the growth medium after 48 h under GLC(+)DARK-LIGHT condition. The (three replications) panel (A) is for WT and (B) for DprqR. In principle, glucose is oxidized to gluconic acid and hydrogen peroxide by glucose oxidase. Hydrogen peroxide then reacts with reduced o-dianisidine in the presence of peroxidase to form a brown colored oxidized o-dianisidine. Oxidized o-dianisidine reacts with sulfuric acid to form a more stable pink colored product. The intensity of the pink color measured at 540 nm is proportional to the original glucose concentration. Thus, the pink color indicates the presence of glucose in WT, whereas no glucose is remained in the medium from DprqR culture.

Supplementary Table S1: The table represents non-targeted metabolic profiling and comparison between DprqR and WT under GLC(+)DARK-LIGHT condition.

Metabolites	Fold Change	P-value	Significance
Dodecanol	5.451781923	0.0243	*
Guanosine	5.259631087	0.0025	*
Mannitol	4.734802839	0.0172	*
Isocitric acid	4.656323724	0.001	***
2-Monopalmitin	4.540057441	0.0221	*
Beta-Mannosylglycerate	4.241111152	0.0004	***
**Methionine**	**4.178951955**	**0.0003**	***
**Isoleucine**	**4.12508114**	**0.0126**	*
linolenic acid	3.781702393	0.0074	**
**putrescine**	**3.496622238**	**0.0243**	*
**Leucine**	3.495717071	0.0069	**
Proline	**3.435972159**	**0.0041**	**
**Serine**	**3.411294322**	**0.0192**	*
L-α-**Alanine**	**3.289842667**	**0.0052**	**
Linoleic acid methyl ester	3.258063014	0.03	*
Tyrosine	3.20954524	0.0116	*
D-Glyceric acid	3.100668139	0.0468	*
**Phenylalanine**	**2.946749354**	**0.0337**	*
**Threonine**	**2.920551457**	**0.0497**	*
D-Threitol	2.754775501	0.0398	*
**L-valine**	**2.684808886**	**0.0399**	*
Ornithine	2.642466585	0.0005	***
Citramalic acid	2.53114301	0.0124	*
N-Acetyl-L-glutamic acid	2.44205097	0.0264	*
Methyl Palmitoleate	2.441661379	0.0241	*
N-formyl-L-methionine	2.378286481	0.0177	*
**Succinic acid**	**2.369362425**	**0.0363**	*
Dodecanoic acid	2.270378299	0.0436	*
Urea	2.252343493	0.0262	*
Hexadecane	2.234889775	0.0343	*
D-**Gluconic Acid**	**2.20397096**	**0.0012**	**
Indole-3-acetamide	2.196160209	0.0004	***
Pyrophosphate	2.052418702	0.0181	*
Noradrenaline	2.005998249	0.0188	*
Glucose-6-phosphate	1.995544793	0.0188	*
Linoleic acid	1.990619188	0.0029	**
3-Hydroxypropionic acid	1.907964575	0.0399	*
2-Amino-2-norbornanecarboxylic acid	1.821033505	0.0383	*
Lactose	1.807767394	0.0217	*
Maltose	1.755695071	0.0369	*
**Reduced Glutathione**	**1.752268178**	**0.031**	*
1-Monopalmitin	1.739619253	0.0116	*
3-Phenyllactic acid	1.614316524	0.0416	*
L-Sorbose	1.599665242	0.0344	*
Pelargonic acid	1.560784886	0.0248	*
**D-Ribose**	**1.547752606**	**0.0264**	*

Metabolites	Fold Change	P-value	Significance
2-Furoic Acid	1.520792195	0.0046	**
Oxoproline	0.970756973	0.0213	*
**Myo-inositol**	**-1.913584201**	**0.0099**	**
Inosine 5'-monophosphate	-2.119275926	0.0008	**
Glycerol-3- phosphate	-2.722441195	0.0428	*
Trehalose	-3.830179265	0.0016	**
**Nicotinic acid**	**-4.14368241**	**0.0036**	**

The fold changes were calculated after DprqR/WT from metabolite detected by GC-TOF-MS, "-"represent down regulation, without any sign is up-regulation, "*" represent significance in t-test where p-value of >0.05 were calculated as significant, the more the significant in t-test increased the number of * sign. Metabolites previously describe as marker [1] during ROS response/ acclimation is marked as 'bold'.

Supplementary Table S2: List of metabolites changes significantly in DprqR compare with WT under GLC(+)DARK condition.

Metabolites	Fold Change	P-value	Significance
Glucose-6-phosphate	3.34966874	0.0204	*
2-Monopalmitin	3.234048698	0.023	*
3-Methylglutaric Acid	2.620424042	0.0076	**
**L-Rhamnose**	**2.419998548**	**0.045**	*
3-Hydroxypropionic acid	1.758730493	0.0018	**
Hypoxanthine	1.307439174	0.0056	**
Guanine	1.226606968	0.0111	*
2-Furoic Acid	1.048495516	0.0337	*
Elaidic acid	1.015768158	0.0086	**
Phosphate	0.826089585	0.0486	*
Cytidine-monophosphate	0.744416003	0.0047	**
Adenine	0.692818329	0.0321	*
3-Hydroxypyridine	0.558536558	0.0052	**
O-Phosphorylethanolamine	0.261880042	0.0016	**
Glycerol-3- phosphate	-1.81031618	0.0064	**
Trehalose	-2.228314205	0.005	**
Sorbitol	-5.887136554	0.0051	**

The fold changes were calculated after DprqR/WT from metabolite detected by GC-TOF-MS, "-"represent down regulation, without any sign is up-regulation, "*" represent significance in t-test where p-value of >0.05 were calculated as significant, the more the significant in t-test increased the number of * sign. Metabolites previously describes as marker [1] during ROS response/ acclimation are marked as 'bold'.

Supplementary Table S3. List of Primers used for vector construction.

pUC95U-cpcKana-95D	
Xba1-D95UF	TGCTCTAGATCGCCTCGTCCAATTTGTT
Kpn1-D95UR	GGGGTACCTAAGCCCTAACCCAATGCC
Sac1-D95DF	CGAGCTCGGTCATTGCCACTGCTCTG
EcoRI-D95DR	CGGAATTCGGACCTCCGACCCAAAGTA
Kpn1-CpcBF	GGGGTACC GTTATAAAATAAACTTAACAAATCTAT
CpcBR	AATTAATCTCCTACTTGACTGTTTTCGTTCCACTGAGCGT
KanaF	ACGCTCAGTGGAACGAAAAC
Sac1-KanaR	CGAGCTCTTATTAGAAAAACTCATCGAGCATC
pBL11U-psbA2-slr0895-His-cpcSmrR-11D	
Pst1-CpcF	AACTGCAGGTTATAAAATAAACTTAACAAATCTAT
smr-CpcR	**CTTCGGCGATCACCGCTTCCCTCAT**AATTAATCTCCTACTTGACT
SmrF	ATGAGGGAAGCGGTGATCG
BamHI-SmrRR	CGCGGATCCTTATTTGCCGACTACCTTGGTGA
Xho1-PsbA2F	CCGCTCGAGAG ATATCGCGTGCAAGGCCCAGTGAT
20nt95-PsbA2R	**ACGAAGCCTTTTTCCAGAAACCA**TTTGGTTATAATTCCTTAGTTCA GATTGGAACTGAC
C-His 95F	ATGGTTTCTGGAAAAAGGCTTC
Pst1-CHis 95R	AAAACTGCAG*CTACTA*GTGGTGGTGGTGGTGGTGTAGCTGGGCCAG TTTCAGA
BamHI-1311DF	CGGGATCCCTGGGCTGATGTATTGAACC
Spe1-1311DR	CGGACTAGTTCCACCAGTTCCAACAAATC
Kpn1-1311UF	GGGGTACCGAATCGCTTCCAATAGTCGTAG
Xho1-1311UR	CCGCTCGAGATTTTTTGGTCACATTGTCCCT
pBL95U-cpcKana-96D	
Sal196DF	ACGCGTCGACTTCCCGTTGCTAGATCTTAATCC
Kpn196DR	CGGGGTACCCCCAAATCCACGCTGGTAAA
Xba195UF	TGCTCTAGAAACCCGATTATAGTCAGCGATGG
Pst195UR	AAAACTGCAGGTCGGTCGGAAGTCAAGTTCAC
Pst1CpcKF	AAAACTGCAGGTTATAAAATAAACTTAACAAATCTAT
Sal1CpcKR	ACGCGTCGACTTATTAGAAAAACTCATCGAGCATC
pBL11U-psbA2-slr0895-His-cpcKana-11D	
Pst1CpcKF	AAAACTGCAGGTTATAAAATAAACTTAACAAATCTAT
BamHI-CpcKR	CGGGATCCTTATTAGAAAAACTCATCGAGCATC

The underlined sequence indicates restriction site of restriction enzyme (with protection base) added with primer. The italic is the sequence of His-tag added with primers. The bold and underlined are complementary sequence for fusion PCR.

Supplementary Table S4. List of primers used for qRT-PCR and Chip-PCR in this study.

qRT-PCR Primers	
slr1843F	TTCGTGAGCAGATGCGTAAGGG
slr1843R	CTCGATTGCCTCTGGTGTTACG
gndF	CTTTCCCATCGCCGTGTTTAAC
gndR	ATTACAAGGATTTTACGGGGGC
pfkB1F	CCACCCAGGGACTAATGGAAAA
pfkB1R	GGAAAAGCGAACGGATTACCCT
rnpBF	TCACAAACCACAGCGGCCTA
rnpBR	GGGCGTTACCCAGCAAGTTT
Hik8F	TTCTGTGCCGTTGCAGTTTTTA
Hik8R	ACGAGGTGGGGATGTTTACTGATTT
Rre37F	GCCCGCCAGGCATTTAATAAC
Rre37R	TGTGTCCTTGGCGGAAAGAATAAA
SigEF	AGACACTGTGGGGGCTTTTTTTA
SigER	CGCTGACCAACAATCGGATTTGT
Hik31F	TCAATCTATCCCGTTGGCGTTTA
Hik31R	CCAATGATCCTGGGAAGTCATTTCAT
gap1F	GTGACCGGGTCATTTTTTTGCTA
gap1R	ATAGGCCAGGTTAGAGGCTGGAA
gap2F	CGCACCGATAGCCAGTTAGAAGTA
gap2R	TCCGGTCGGAAACACATTTAATAG
Slr0896F	GGCATTTATGATGACTGGGGTGG
Slr0896R	ACCCTGACGAGTTAATTGCCCAA
sodBF	GCCGCCTACGTTAACAATTTCA
sodBR	CACCGGGCTTCATGCAATT
Chip PCR primer	
96F1	GCGACATTTTTGGCAAGAGTGC
96R1	CAGTTTCAGAGCAGTGGCAATG
96F2	GTTTTGCTCCCCTGGAAGAC
96R2	TCTAAAGATATTCCCTAGAGCCACC
96F3	GTTCTTTGGGTCAATGCTATGACA
96R3	CCCATCATGACCGTATCGAC

Supplementary Table S5: Analysis of Pyridine nucleotide detected by LC-MS.

Nucleotide	Cell type	HT	DIMHL
NADPH/NADP	WT	1.442147702	0.838247
	DprqR	0.977599185	0.715736808
ATP/ADP	WT	0.863158955	1.567981411
	DprqR	0.861609043	1.904252999
ATP/NADPH	WT	594.0792327	1346.001532
	DprqR	1086.612716	3354.619984

Reference

1. Noctor, G., Lelarge-Trouverie, C. & Mhamdi, A. The metabolomics of oxidative stress. *Phytochemistry* **112**, 33-53 (2015).
